# Unusual Rearrangements in Cyclohex-3-ene-1-carboxamide Derivatives: Pathway to Bicyclic Lactones

**DOI:** 10.1021/acsomega.4c02183

**Published:** 2024-05-14

**Authors:** Ozlem Gundogdu, Sertan Aytaç, Ertan Şahin, Yunus Kara

**Affiliations:** † Department of Food Technology, Kaman Vocational School, Ahi Evran University, Kirsehir 40100, Turkey; ‡ Department of Chemistry, Faculty of Science, Atatürk University, Erzurum 25240, Turkey

## Abstract

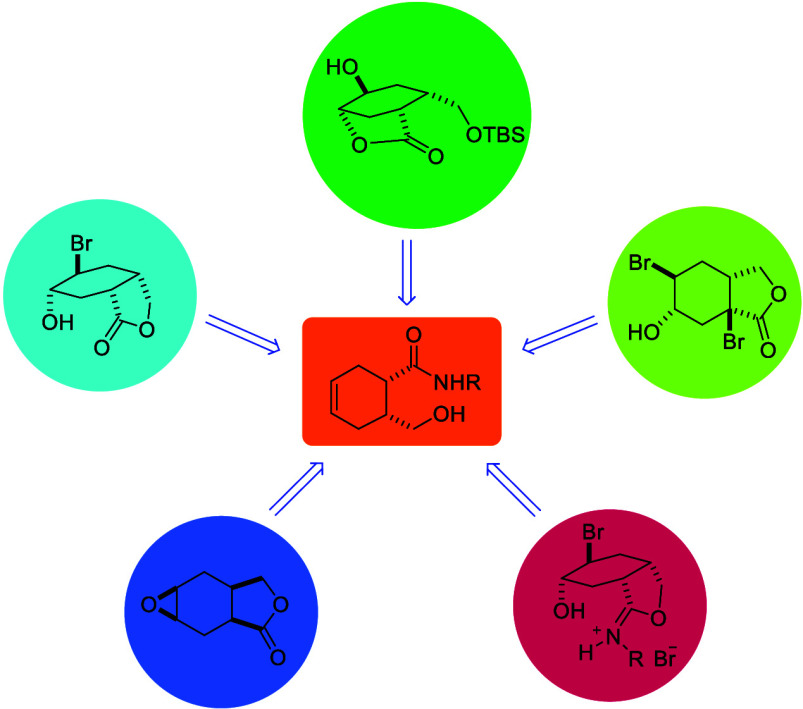

The synthesis of new bicyclic lactone derivatives was carried out starting from 2-methyl/phenyl-3*a*,4,7,7*a*-tetrahydro-1*H*-isoindole-1,3(2*H*)-dione. 6-(Hydroxymethyl)-*N*-methyl/phenylcyclohex-3-ene-1-carboxamide derivatives were obtained from the reduction of tetrahydro-1*H*-isoindole-1,3(2*H*)-diones with NaBH_4_. Bromination and epoxidation reactions of both compounds were examined, and the structures of the resulting products were determined by spectroscopic methods. Substituted bicyclic lactone compounds, which are interesting rearrangement products in both bromination and epoxidation reactions, were obtained. In particular, hydroxymethyl (−CH_2_OH) and amide (−CONHR) groups attached to the cyclohexene ring in the bromination and epoxidation reactions were found to be effective in product formation. As a result, a new and applicable method was developed for the synthesis of bicyclic lactone derivatives.

## Introduction

Glycosidases, also known as glycoside hydrolases, represent a crucial group of enzymes that facilitate the hydrolytic cleavage of glycosidic bonds.^1−4^ These enzymes are prevalent across a broad range of organisms, including microorganisms, plants, and animals, and have significant applications in various fields, such as biotechnology, the food industry, and pharmacology. α/β-Glucosidases specialize in breaking down α- or β-linkages at the anomeric center of a glucose fragment. They play essential roles in numerous biochemical pathways that are linked to various metabolic disorders and diseases including diabetes, viral and bacterial infections, lysosomal storage disorders, and cancer.^1,2^ Consequently, considerable research has been dedicated to developing effective glucosidase inhibitors. These inhibitors are typically designed to mimic a substrate, a transition state, or an enzyme reaction product. As such, various polyhydroxylated cyclic compounds, which are five- or six-membered, are explored as monosaccharide structural analogues and potential inhibitors of glycosidases.^5−8^

Brazdova et al.^9^ developed and tested a variety of cyclohexanecarboxylic acids featuring diverse substitution patterns, substituent configurations, and varying lengths of *n*-alkyl groups R to investigate their impact on inhibitory activity. These molecules, characterized by a six-membered-ring structure adorned with multiple hydrophilic groups, align with the common structural features of many carbohydrate mimetics. The introduction of alkyl groups aimed to bolster the inhibitory effect, a phenomenon similarly noted in prior studies with lipophilic groups on compounds such as 1-deoxynojirimycin derivatives, other iminosugars, alkyl glycosides, and various additional glycosidase inhibitors.^9^

**Scheme 1:**
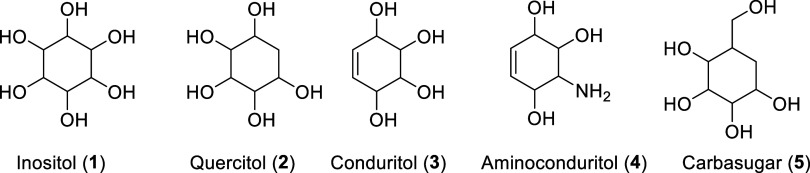
Structures of Commonly Known Cyclitol Derivatives

Cyclitols make up a group of cycloalkanes that are characterized by the presence of at least three hydroxyl groups, with each one attached to a distinct carbon atom in the ring structure. Cyclitol is also used for similar molecules containing one or more double bonds in the ring with various functional groups replacing the hydrogen atoms in such a molecule. Cyclitols are classified as inositols (**1**), quercitols (**2**), conduritols (**3**), and aminoconduritols (**4**) (Scheme 1).^10−12^ Cyclitol derivatives are used as sweeteners, glycosidase inhibitors, antidiabetic, antifungal, anticancer, antibiotic, and antiviral reagents.^5−8^ On the other hand, carbasugars are cyclitol derivatives with significant biological activity. Their synthesis as alternative glycosidase inhibitors has attracted great interest in pharmaceutical and organic chemistry in recent years.^4,13−16^

On the other hand, syntheses of cyclitol derivatives containing different functional groups from bicyclic or tricyclic systems have been reported in the literature.^17−20^ Two main reactions, epoxidation and bromination, were used in the synthesis of cyclitol derivatives, and lactone derivatives containing a five-membered ring were synthesized.

**Scheme 2:**
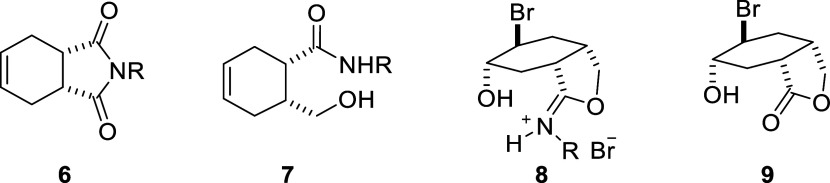
Structures of Compounds **6**, **7**, **8**, and **9**

Recently, we were interested in the synthesis of *y*-hydroxycarboxamide **7** from the reduction of bicyclic imide, from which tetrasubstituted cyclohexane derivatives can be prepared by manipulating alkene double bonds. In this context, we were surprised by the formation of rearrangement product **8**, which was formed upon bromination of *y*-hydroxycarboxamide **7**. Hydrolysis of iminium **8** gave bicyclic lactone **9** (Scheme 2).^21^

In light of these findings, we decided to investigate the bromination and epoxidation reactions of cyclohexene derivatives containing *N*-methyl and *N*-phenyl-carboxamide units. Here, we report the alternative strategy developed for the synthesis of tetrasubstituted cyclohexane derivatives. We also discuss the mechanism of formation of the products in the bromination and epoxidation reactions.

## Results and Discussion

For the synthesis of tetrasubstituted cyclohexane derivatives, we used bicyclic imide compounds **6a** and **6b** obtained in two steps from 3-sulfolene as the key compound. When imide derivatives are reduced with metal hydrides, product formation in these reactions depends on parameters such as the amount of reagent used, type of reagent, and reaction time. Therefore, the conversion of both imide compounds **6a** and **6b** to the corresponding carboxamide compounds **7a** and **7b** was carried out under reaction conditions optimized in previous studies.^22^

**Scheme 3:**
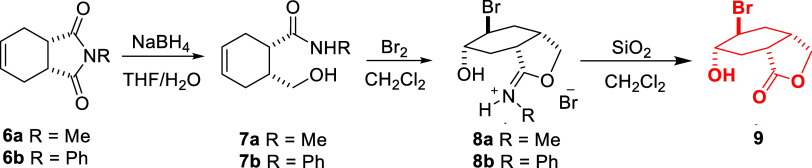
Synthesis and Their Reactions of γ-Hydroxycarboxamide Derivatives **7**

In this context, the imide compounds **6a**–**b** were reacted with 2 equiv of NaBH_4_ in THF/H_2_O (1:1) and the reaction was quenched after 6 h (Scheme 3). The products were purified, and their structures were elucidated by spectroscopic methods. The carboxamide compounds **7a**–**b** are formed as a result of imide group reduction, with the primary alcohol (−CH_2_OH) and amide (−CONHR) groups and the peaks belonging to both groups are clearly seen in the ^1^H and ^13^C NMR spectra (Figures S1, S2, S4, S5).

After synthesizing carboxamide **7** containing three functional groups (−CH_2_OH, −CONHR, and double bond), we studied the bromination and epoxidation reactions of these compounds. One of the aims of studying these reactions is to synthesize tetrasubstituted cyclohexane derivatives, and the second is the expectation that interesting rearrangement products may arise in both reactions due to the effect of bonded groups in cyclic systems. We very recently studied the bromination of the carboxamide compound **6a**. In this reaction, an iminium salt was obtained by the action of both amide (−CONHR) and alcohol (−CH_2_OH) groups attached to the ring, and the mechanism of product formation was also discussed. Subsequently, hydrolysis of the iminium group yielded the lactone compound. It is very interesting that although only Br_2_ was used in the reaction, halohydrin (HOBr) was added to the double bond in the final product. Because Br_2_/H_2_O or HOBr was not used as a reagent. In this context, when the reaction steps were evaluated, an intramolecular hydroxyl (−OH) group transfer occurred in the reaction. This result makes the applied method even more important (Scheme 3).^21^

**Scheme 4:**
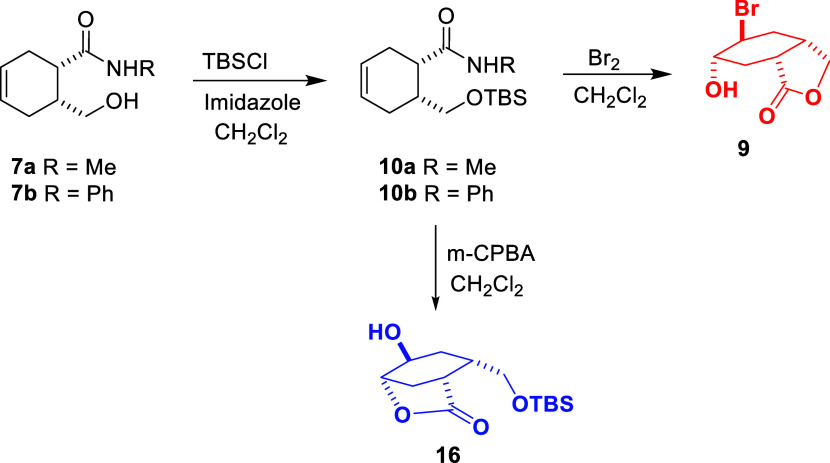
Synthesis and Their Reactions of Silyl Ether Compounds

In this context, considering what kind of product can be obtained by protecting the alcohol group that causes lactone formation, the alcohol group was converted to the corresponding silyl ether with *tert-*butyldimethylsilyl chloride to give compound **10** (Scheme 4).

**Scheme 5:**
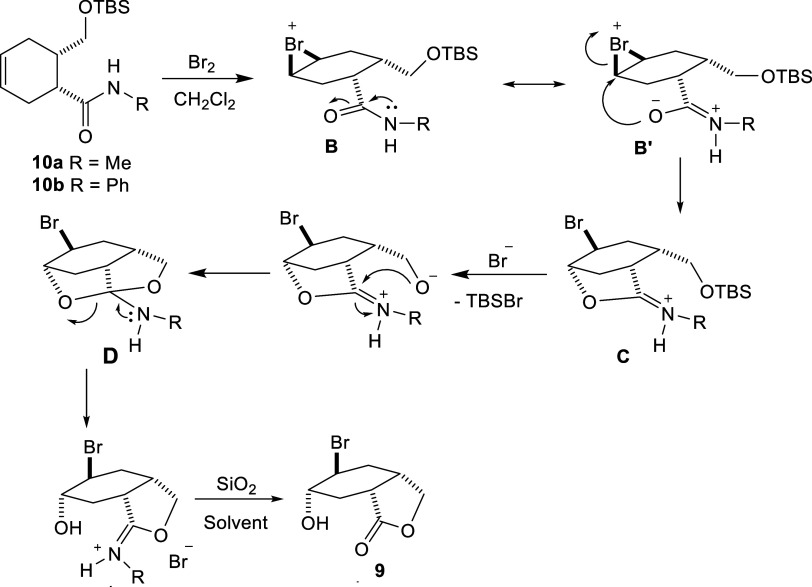
Bromination of Carboxamide Derivative **10** Containing Silyl Ether Group

The bromination reaction of compound **10** containing the protected alcohol group was investigated. The resulting lactone compound obtained in the previous reaction was synthesized. The formation of the same lactone compound can be explained by the fact that the reaction does not proceed similarly to compound **7** as a result of the removal of the silyl group in compound **10** under the reaction conditions. It is predicted that the reaction takes place through the mechanism given in Scheme 5.^23−25^

**Figure 1:**
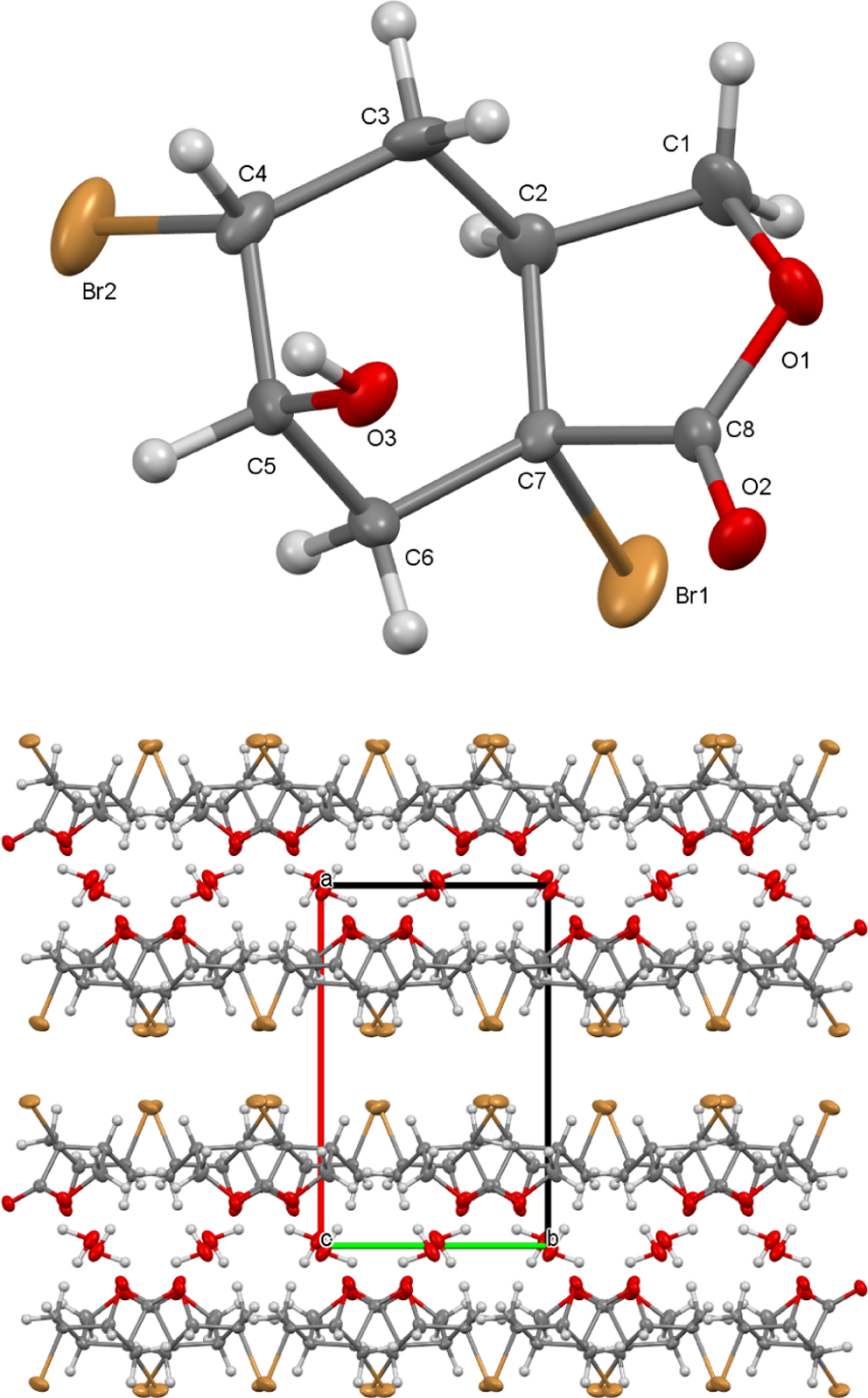
(Top) X-ray structure of molecule **12**. Thermal ellipsoids are drawn at the 40% probability level. (Bottom) Trapped aqua molecules between layers of molecule **12** and the unit cell are viewed down along the *c*-axis.

**Scheme 6:**
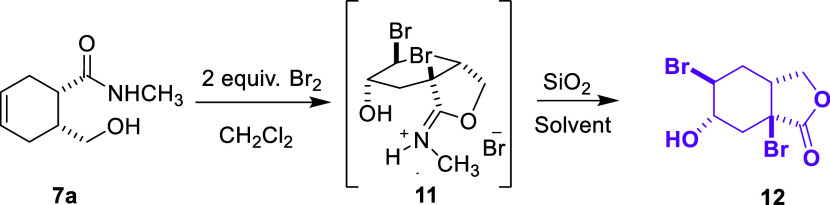
Bromination of Carboxamide Derivative **7a** with Excess Bromine

When 2 equiv of bromine was used for the bromination reaction, a second bromine atom was found to be attached to the molecule and the exact structure of the molecule was determined by X-ray diffraction (Figure 1). Based on the molecular structure, it is predicted that the second bromine atom is bound to the molecule *via* the enol form formed by the carbonyl group (Scheme 6).

The exact conformation and structures of the 5,7*a*-dibromo-6-hydroxyhexahydro-2-benzofuran-1(3*H*)-one hydrate (**12**) was confirmed by X-ray diffraction analysis. Molecule **12** is in racemic form, and only one enantiomer is seen in the asymmetric unit (Figure 1). The molecule **12** was crystallized in the monoclinic *P*2_1_/*c* space group. It consists of fused cyclohexane and heterocyclic furan rings with substituted bromine, −OH, and carbonyl oxygen atoms. There is also 1 mol of aqua molecule in this structure. Here, the cyclohexane ring is in the chair conformation and has the most stable conformation. In this cycle, C–C single bonds are in the range of 1.494–1.535(3) Å. Bromine atoms are in *cis* position and the bond lengths of C4–Br2 and C7–Br1 are 1.963 and 1.982 Å, respectively. Aqua molecules are trapped by hydrogen bonds between strings of molecules **12**. These paramount H-bonds are as follows: O3–H···O4 [D···A = 2.651(3)Å], O4–H···O2 [D···A = 2.891(3)Å] and O4–H···O3 [D···A = 2.785(3) Å]. Finally, we can state that the furan ring is in an envelope form. Maximum deviation of C2 atom from meanplane C1/O1/C8/C7 is 0.203 Å.

**Scheme 7:**
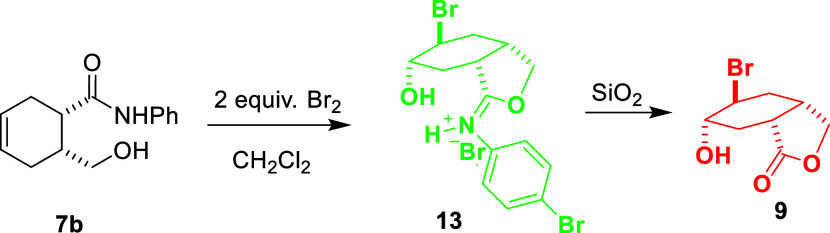
Bromination of Carboxamide Derivative **7b** with Excess Bromine

On the other hand, bromination was carried out in systems containing a phenyl group instead of a methyl group attached to the nitrogen atom in the amide group (Scheme 3). A similar rearrangement product **8b** was also observed in the phenyl-containing compound. As a result, lactone compound **9** was obtained through the hydrolysis of compound **8b**. However, in the reaction of compound **7b** containing phenyl group with 2 equiv. bromine, unlike compound **7a**, the second bromine atom was found to be attached to the phenyl ring in the molecule (Scheme 7). This result was confirmed by the ^1^H NMR spectrum of the crude product (Figures S13 and S14). Since the molecule was hydrolyzed during the purification process, lactone **9** was obtained as a result of hydrolysis. According to the ^1^H NMR spectrum analysis of compound **13**, the bromine atom is not bound to the cyclohexane ring, since there is no change in the number of protons and their signal groups in the cyclohexane ring. On the other hand, the differences in the number of phenyl ring protons and their chemical shift values in molecule **13** indicate that the second bromine atom is bound to the phenyl group. In particular, the fact that phenyl protons change from a multiplet appearance before the reaction to an AB system after the reaction shows that the second bromine atom is bonded to the *p*-position of the phenyl ring in molecule **13**.

**Scheme 8:**
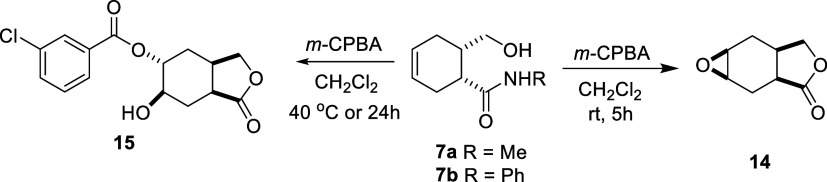
Synthesis of Hexahydrooxireno[2,3-*f*]isobenzofuran-3(1*aH*)-one (**14**) and 6-Hydroxy-1-oxooctahydro-2-benzofuran-5-yl-3-chlorobenzoate (**15**)

Interesting rearrangement products were obtained in bromination reactions, and the structures and formation mechanisms of these products were discussed. Next, we decided to examine the epoxidation reactions of carboxamides for which rearrangement products were also expected. First, we carried out the epoxidation reaction of carboxamide **7a** with *m*-CPBA in methylene chloride. As a result of the reaction, epoxy lactone compound **14** was obtained (Scheme 8).

After separation, ^1^H and ^13^C NMR spectroscopic data showed that a product different from expected was formed (Figures S18 and S19). In the ^1^H NMR spectrum of the product, CH_2_ protons in the lactone ring give the AB system at δ = 4.40 and 4.03 ppm. In addition, the epoxide ring protons resonate in the form of the multiplet at δ = 3.23–3.18 ppm due to the asymmetry in the molecule. These protons are split into neighboring CH_2_ protons. In the ^13^C NMR spectrum of compound **14**, the carbonyl carbon and –OCH_2_– carbon in the lactone ring resonate at δ = 170 and 60 ppm, respectively. Additionally, 8 lines in the ^13^C NMR spectrum are in complete agreement with the structure of the molecule. The exact stereochemistry of the epoxide ring in **14** was later confirmed by single-crystal X-ray analysis of the epoxide ring opening product **15**.

When the reaction mechanism is examined, depending on the structure of the lactone molecule **14**, three different reactions occur. These are epoxide, amide hydrolysis, and lactone formation. Amide hydrolysis and lactonization can also occur in a single step. Phenyl isomer **7b** also gave the same *syn*-epoxide **14**.

**Figure 2:**
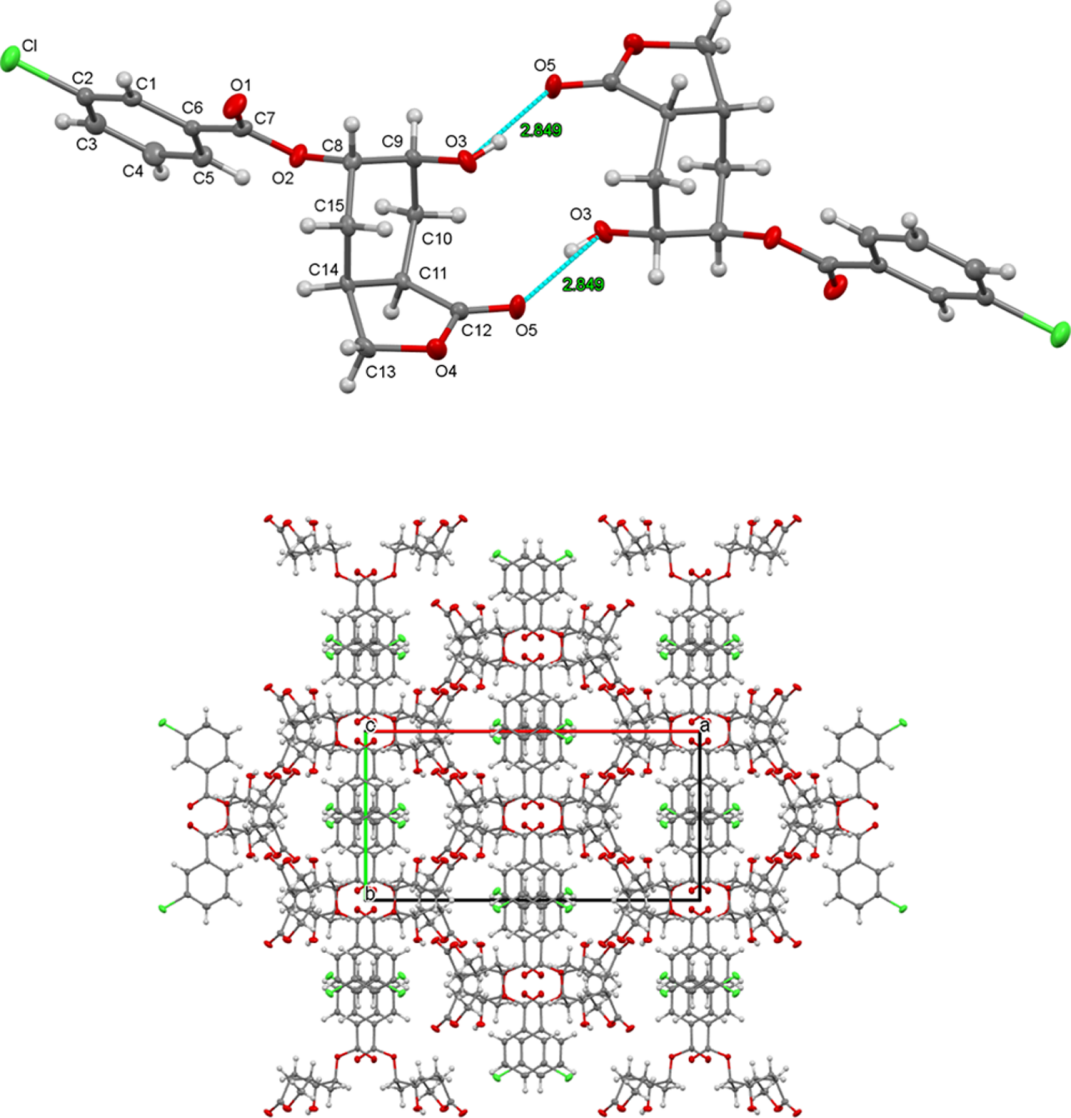
(Top) X-ray structure of dimeric molecule **15**. Thermal ellipsoids are drawn at the 40% probability level. (Bottom) Stacking motif and the unit cell viewed down along the *c*-axis.

When the reaction time was prolonged or the reaction temperature was increased to 35–40 °C, the epoxide opening product **15** was obtained (Scheme 8). Compound **15** was crystallized from CH_2_Cl_2_–hexane solvent mixture and X-ray analysis was performed (Figure 2).

The exact conformation and structures of 6-hydroxy-1-oxooctahydro-2-benzofuran-5-yl-3-chlorobenzoate (**15**) were confirmed by X-ray diffraction analysis. Molecule **15** is in racemic form, and only one enantiomer is seen in the asymmetric unit (Figure 2). The molecule **15** crystallizes in monoclinic space group *C*2/*c* with eight molecules in the unit cell. The core structure is again a fused cyclohexane and lactone ring, and −OH, carbonyl, and *m*-CPBA groups are attached to the core unit. Two enantiomers form a dimeric structure with O3–H–O5 [D···A = 2.849(3)Å] hydrogen bonding (Figure 2). The cyclohexane ring in the chair conformer and for this cycle C–C single bonds are in the 1.508–1.528(3) Å range. Here again, we can state that the furan ring is in the envelope form. Maximum deviation of C14 atom from meanplane C12/O4/C13/C11 is 0.224 Å. The racemic nature of the molecules significantly affects the supramolecular structures and crystal lattice motifs (Figure 2).

We expected the formation of the *anti*-epoxy isomer due to the asymmetry in molecule **7**. However, X-ray analysis of epoxy ring opening product **15** showed that the *syn-*epoxy isomer was formed in the epoxidation reaction. In our previous studies, we expected the formation of the *anti*-epoxide isomer due to the steric effect but found that the *syn*-isomer was formed in much larger amounts.^26,27^ This result, the further formation of the *syn*-isomer, was explained by the dipole–dipole interaction between the imide ring and the per acid in the compound. Similar to these examples, an important factor in the formation of the *syn*-epoxy isomer from the epoxidation of compound **7** is the intermolecular interactions (hydrogen bonding, etc.) of the per acid with the alcohol and amide groups in the molecule, resulting in the per acid approaching the molecule from the same side as the alcohol and amide group to form the *syn*-isomer.

**Scheme 9:**
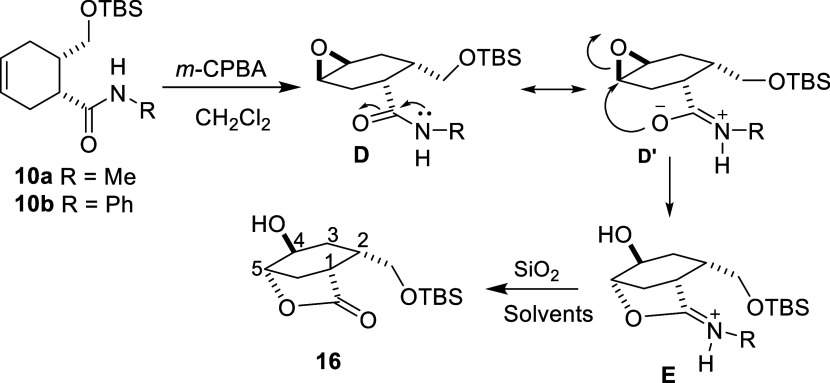
Epoxidation of the Carboxamide Derivative **10**

In order to investigate the effect of the alcohol group on isomer formation in epoxidation reactions, the OH group was protected with TBSCl as in the bromination reaction, and the epoxidation reaction was carried out. After purification, it was determined by spectroscopic methods that a new rearrangement product was formed from the epoxidation of the carboxamide molecule containing the protected alcohol group (Scheme 9). In the ^1^H NMR spectrum of compound **16**, signal groups belonging to the epoxide and amide group are not present (Figures S24 and S25). This indicates that the epoxide ring is opened, and the amide group is hydrolyzed. In addition, −CH_2_OTBS protons give the AB system at δ = 3.43 and 3.33 ppm. H_4_ and H_5_ protons in the six-membered ring resonate at δ = 4.58 and 4.15 ppm, respectively. The presence of 14 lines in ^13^C NMR is in agreement with the structure.

When the products formed in the epoxidation reaction of compounds **7** and **10** are compared, *syn*-isomer is formed in compound **7** (free alcohol group) while *anti*-isomer is formed in compound **10** (protected alcohol group) and the carboxylic acid group formed by hydrolysis of the amide group attacks from behind and causes the epoxide ring to open. This result supports our suggestion of explaining the *syn*-isomer. Thus, two different lactones were obtained from the epoxidation of compound **7** and compound **10**. However, since the amide group is hydrolyzed in the reaction medium, the same lactone **16** is formed from both compounds **10a** and **10b**.

## Conclusions

In conclusion, we examined the bromination and epoxidation reactions of cyclohex-3-ene-1-carboxamide derivatives **7** and **10** obtained from the reduction of a bicyclic imide with NaBH_4_. We obtained the same lactone **9** from the bromination reaction of both compounds and discussed the reaction mechanism. In addition, in compound **7**, we examined the reaction of two compounds with methyl and phenyl groups attached to the nitrogen atom with excess bromine. First, we synthesized isobenzofuran derivative **12**, which has a second bromine atom attached to the bridgehead, from the reaction of the compound with a methyl group attached to the nitrogen atom with excess bromine. In this reaction, the attachment of the second bromine atom to the ring can be defined as the α-bromination reaction of the carbonyl group. Second, we performed the reaction of the compound in which the phenyl group is attached to the nitrogen atom with excess bromine. As a result of this reaction, we determined that the second bromine atom was bound to the *p*-position of the phenyl ring, as a result of the electrophilic substitution reaction.

On the other hand, we obtained two different lactone compounds from the epoxidation of compounds **7** and **10**. We determined that intermolecular interactions (hydrogen bonds, etc.) play a role in the formation of both lactones. We postulate that the most important reason for reactions to proceed through the proposed mechanism is the canonical forms of the amide group.

As a result, we developed a new lactone synthesis method by bromination and epoxidation reactions depending on the groups in the ring.

## Experimental Section

### General Information

All reagents used were commercially available unless otherwise specified, and all solvents were distilled before use. Melting points were measured with a Gallenkamp melting point device. ^1^H NMR and ^13^C NMR spectra were recorded on a Bruker (400 MHz ^1^H, 100 MHz ^13^C) or Varian (400 MHz ^1^H, 100 MHz ^13^C). Chemical shift values (δ ppm) are reported using tetramethylsilane (δH 0.00), CDCl_3_ (δC 77.00), and CD_3_OD (δC 49.15). The ^1^H NMR spectra are reported as follows δ (number of protons, multiplicity, coupling constant *J* Hz). Multiplicities are indicated by s (singlet), d (doublet), t (triplet), q (quartet), dd (doublet of doublet), m (multiplet), and br-s (broad singlet). High-resolution mass spectrometry (HR-MS): Electron spray technique (M+/M−) from the solution. in MeOH (Waters LCT Premier XE UPLC/MS TOF (Manchester, U.K.)).

#### Synthesis of 6-(Hydroxymethyl)-*N*-methyl/Phenylcyclohex-3-ene-1-carboxamide (**7**)

To a stirred solution of isoindole-1,3-dione **6** (2.20 mmol) in THF/H_2_O (20 mL, 1:1) was added NaBH_4_ (4.40 mmol) at 0 °C for 5 min. After stirring at room temperature for 6 h, saturated NH_4_Cl was added to the reaction mixture. The organic layer was separated and the aqueous layer was extracted 3 × 10 mL with EtOAc. The combined organic layer was dried over Na_2_SO_4_ and the solvent was removed in vacuo. The residue was purified by silica gel column chromatography (Hexane/EtOAc: 40/60 → 0/100) to afford **7**.

##### 6-(Hydroxymethyl)-*N*-methylcyclohex-3-ene-1-carboxamide (**7a**)

White solid (95% yield). mp. 88–90 °C. **^1^H NMR** (400 MHz, CDCl_3_) δ 6.62 (s, 1H), 5.66 (d, *J* = 2.5 Hz, 2H), 3.97 (brs, 1H), 3.68–3.32 (m, 3H), 2.74 (s, 3H), 2.42–2.04 (m, 4H), 1.84 (dd, *J* = 16.3, 4.6 Hz, 1H). **^13^C NMR** (100 MHz, CDCl_3_) δ: 173.42, 127.14, 125.00, 64.09, 41.91, 37.16, 27.22, 26.33, 25.62. **HRMS**: (ESI), *m*/*z*: Calcd. for [M + H]^+^ C_9_H_15_NO_2_: 170.1103; found: 170.1174.

##### 6-(Hydroxymethyl)-*N*-phenylcyclohex-3-ene-1-carboxamide (**7b**)

White solid (96% yield), mp. 155–157 °C. ^**1**^**H NMR (400 MHz CDCl**_**3**_**):** δ 7.96 (brs, 1H, N–H), 7.52–7.47 (m, 2H), 7.36–7.30 (m, 2H), 7.16–7.10 (m, 1H), 5.85 (brs, 2H), 3.75–3.62 (m, 2H), 3.07–3.02 (m, 1H), 2.61–2.26 (m, 3H), 2.24–2.12 (m, 1H), 1.98–1.88 (m, 1H). ^**13**^**C NMR (100 MHz, CDCl**_**3**_**)** δ: 173.41, 137.61, 129.02, 127.35, 124.98, 124.51, 120.13, 64.24, 41.96, 37.23, 26.36. **HRMS**: (ESI), *m*/*z*: [M + H]^+^ C_14_H_17_NO_2_ calcd. 232.1259; found 232.1313.

#### (1*S*,6*R*)-6-(((*tert*-Butyldimethylsilyl)oxy)methyl)-*N*-methyl/Phenylcyclohex-3-ene-1-carboxamide (**10**)

In a 50 mL single-neck flask, the starting compound **7** (2.16 mmol) and imidazole (4.32 mmol) were dissolved by adding 30 mL of CH_2_Cl_2_ and stirred for 5 min at room temperature in N_2_ atm. Then, *tert*-butyldimethylsilyl chloride (2.38 mmol) dissolved in 10 mL of CH_2_Cl_2_ was added to the mixture and stirred for 16 h at room temperature. When the reaction was completed, 10 mL of a saturated NH_4_Cl solution was added. The mixture was extracted with CH_2_Cl_2_ (3 × 20 mL). The organic phase was washed again with a saturated NH_4_Cl (20 mL) solution. It was dried over Na_2_SO_4_. The crude product was purified on a silica gel column in a 20% EtOAc/*n*-hexane solvent system.

##### (1*S*,6*R*)-6-(((*tert*-Butyldimethylsilyl)oxy)methyl)-*N*-methylcyclohex-3-ene-1-carboxamide (**10a**)

White solid (92% yield), mp. 73–74 °C. ^**1**^**H NMR (400 MHz, CDCl**_**3**_**)** δ 6.03 (s, 2H), 5.66 (s, 4H), 3.52 (t, *J* = 9.0 Hz, 4H), 2.84–2.63 (m, 8H), 2.36–1.83 (m, 11H), 0.84 (s, 16H), −0.00 (s, 9H). ^**13**^**C NMR (100 MHz, CDCl**_**3**_**)** δ: 174.41, 125.87, 125.43, 77.34, 77.02, 76.70, 64.14, 40.65, 37.64, 26.25, 26.16, 26.01, 25.89, 18.22, −5.41. **HRMS**: (ESI), *m*/*z*: [M + H]^+^ C_15_H_29_NO_2_Si calcd. 284.1968; found 284.2040.

##### (1*S*,6*R*)-6-(((*tert*-Butyldimethylsilyl)oxy)methyl)-*N*-Phenylcyclohex-3-ene-1-carboxamide (**10b**)

White solid (94% yield), mp. 115–116 °C. ^**1**^**H NMR** (400 MHz, CDCl_3_) δ 7.94 (s, 1H), 7.40 (d, *J* = 7.7 Hz, 2H), 7.27–7.09 (m, 2H), 6.96 (t, *J* = 7.4 Hz, 1H), 5.83–5.55 (m, 2H), 3.63 (dd, *J* = 10.8, 6.8 Hz, 1H), 3.58–3.46 (m, 1H), 3.01–2.81 (m, 1H), 2.39 (d, *J* = 16.7 Hz, 1H), 2.24–2.04 (m, 2H), 2.01–1.73 (m, 2H), 0.84 (s, 8H), −0.00 (s, 5H). ^13^C NMR (100 MHz, CDCl_3_) δ: 171.90, 138.29, 128.92, 125.64, 125.38, 123.77, 119.52, 64.50, 40.46, 38.00, 26.46, 25.95, 25.52, 18.23, −5.24. **HRMS**: (ESI), *m*/*z*: [M + H]^+^ C_20_H_31_NO_2_Si calcd. 346.2124; found 346.2196.

#### Synthesis of 5-Bromo-6-hydroxyhexahydroisobenzofuran-1(3*H*)-one (**9**)

To a stirred solution of carboxamide **10** (1.45 mmol) in dichloromethane (20 mL) was added bromine (1.74 mmol) in an ice bath reaction mixture. The reaction was quenched after 5 min. The solvent was removed in vacuo. It was observed that it transformed into a different product on the silica gel column during purification. The lactone compound **9** was obtained in 95% yield mp: 111–113 °C. ^**1**^**H NMR** (400 MHz, CDCl_3_) δ 4.36–4.23 (m, 2H), 4.12–4.00 (m, 2H), 2.94–2.81 (m, 1H), 2.72–2.62 (m, 1H), 2.52–2.31 (m, 2H), 2.24–2.13 (m, 1H), 2.06–1.96 (m, 1H). ^**13**^**C NMR** (100 MHz, CDCl_3_) δ: 179.15, 77.58, 77.26, 76.94, 71.23, 67.78, 50.61, 36.22, 31.63, 28.42, 24.98. **HRMS**: (ESI), *m*/*z*: [M + H]^+^ C_8_H_11_BrO_3_ calcd. 234.9892; found 234.9965.

#### Synthesis of 5,7*a*-Dibromo-6-hydroxyhexahydroisobenzofuran-1(3*H*)-one (**12**)

To a stirred solution of carboxamide **7** (2.16 mmol) in dichloromethane (20 mL) was added bromine (4.32 mmol) in an ice bath. The reaction was stirred after 10 min. The solvent was removed in vacuo. Lactone compound **12** was obtained in 95% yield by hydrolysis of the product in a silica gel column during purification. White crystal, mp 114–115 °C. ^**1**^**H NMR** (400 MHz, CDCl_3_) δ 4.63–4.53 (m, 1H), 4.22–4.17 (m, 1H), 4.09 (dd, *J* = 6.1, 3.0 Hz, 1H), 3.97 (d, *J* = 9.2 Hz, 1H), 3.09–3.00 (m, 1H), 2.99–2.90 (m, 1H), 2.72 (dd, *J* = 15.1, 2.6 Hz, 1H), 2.40–2.28 (m, 1H), 2.11–2.01 (m, 1H). ^**13**^**C NMR** (100 MHz, CDCl_3_) δ: 174.76, 71.16, 69.77, 55.34, 48.03, 41.70, 36.27, 30.06. **HRMS**: (ESI), *m*/*z*: [M + H]^+^ C_8_H_10_Br_2_O_3_ calcd. 314.9049; found 314.9051

#### Synthesis of Hexahydrooxireno[2,3-*f*]isobenzofuran-3(1*aH*)-one (**14**)

Compound **7** (2.16 mmol) and 77% *m*-CPBA (4.32 mmol) were dissolved in 30 mL of DCM. The reaction was stirred at room temperature for 5 h. The solvent was removed from the evaporator. 10 mL of saturated NaHCO_3_ solution was added to the residue, and the mixture was extracted with DCM (3 × 20 mL). The organic phase was washed again with saturated NH_4_Cl (20 mL) solution. It was dried over Na_2_SO_4_. The crude product was purified on a silica gel column in a 20% EtOAc/*n*-Hexane solvent system, 96% yield. White solid, mp. 95–97 °C. ^**1**^**H NMR** (400 MHz, CDCl_3_) δ 4.36–4.23 (m, 2H), 4.12–4.00 (m, 2H), 2.94–2.81 (m, 1H), 2.72–2.62 (m, 1H), 2.52–2.31 (m, 2H), 2.24–2.13 (m, 1H), 2.06–1.96 (m, 1H). ^**13**^**C NMR** (100 MHz, CDCl_3_) δ 179.15, 77.58, 77.26, 76.94, 71.23, 67.78, 50.61, 36.22, 31.63, 28.42, 24.98. **HRMS**: (ESI), *m*/*z*: [M + H]^+^ C_8_H_11_BrO_3_ calcd. 234.9892; found 234.9965.

#### Synthesis of 6-Hydroxy-1-oxooctahydroisobenzofuran-5-yl-3-chlorobenzoate (**15**)

Starting compound **7** (2.16 mmol) and 77% *m*-CPBA (4.32 mmol) were dissolved in 30 mL of DCM. The reaction was stirred at room temperature for 1 day. The solvent was removed from the evaporator. 10 mL of saturated NaHCO_3_ solution was added to the residue, and the mixture was extracted with DCM (3 × 20 mL). The organic phase was washed again with saturated NH_4_Cl (20 mL) solution. It was dried over Na_2_SO_4_. The crude product was purified on a silica gel column in a 20% EtOAc/*n*-hexane solvent system, 92% yield. Colorless crystal, mp. 154–156 °C. ^**1**^**H NMR (400 MHz, CDCl**_**3**_**)** δ 7.96 (t, *J* = 1.8 Hz, 1H), 7.93–7.88 (m, 1H), 7.59–7.54 (m, 1H), 7.41 (t, *J* = 7.9 Hz, 1H), 5.21 (dd, *J* = 7.4, 4.4 Hz, 1H), 4.32 (dd, *J* = 9.1, 5.2 Hz, 1H), 4.13–4.03 (m, 2H), 2.78 (dtd, *J* = 17.8, 11.2, 6.3 Hz, 2H), 2.28–2.24 (m, 2H), 2.20–2.11 (m, 1H), 2.07–1.97 (m, 1H). ^**13**^**C NMR (100 MHz, CDCl**_**3**_**)** δ 178.94, 164.36, 134.66, 133.34, 131.65, 129.88, 129.54, 127.77, 71.52, 71.47, 65.20, 36.39, 31.34, 26.00, 25.07. **HRMS**: (ESI), *m*/*z*: [M + H]^+^ C_15_H_15_ClO_5_ calcd. 311.0608; found 311.0680.

#### Synthesis 2-(((*tert*-Butyldimethylsilyl)oxy)methyl)-4-hydroxy-6-oxabicyclo[3.2.1]octan-7-one (**16**)

After compound **10** (1.45 mmol) was dissolved in DCM, *m*-CPBA (1.74 mmol) was added. The reaction mixture is monitored by TLC control at room temperature. After 6 h, the reaction was stopped by adding saturated NH_4_Cl. The crude product was extracted with 3 × 20 mL DCM, and the organic phase was separated. The organic phases were dried over Na_2_SO_4_, and the solvent was removed in the evaporator. The crude product was purified on a silica gel column, 84% yield. Yellow viscous. ^**1**^**H NMR** (400 MHz, CDCl_3_) δ 4.58 (t, *J* = 5.0 Hz, 1H), 4.10 (t, *J* = 4.1 Hz, 1H), 3.46–3.38 (m, 1H), 3.32 (dd, *J* = 10.1, 5.8 Hz, 1H), 2.70 (dt, *J* = 22.1, 7.4 Hz, 2H), 2.34–2.10 (m, 3H, OH+2H), 1.78 (dd, *J* = 15.1, 4.9 Hz, 1H), 1.41–1.31 (m, 1H), 0.82 (s, 9H), −0.00 (s, 6H). ^**13**^**C NMR** (100 MHz, CDCl_3_) δ: 177.82, 79.27, 64.79, 64.46, 39.53, 37.16, 31.36, 30.62, 25.88, 18.25, −5.42. **HRMS**: (ESI), *m*/*z*: [M + H]^+^ C_14_H_26_O_4_Si calcd. 287.1600; found 287.1672.

### Crystal Structure Determination

For the determination of the crystal structure, single crystals of molecules **12** and **15** were analyzed using a Rigaku R-AXIS RAPID-S four-circle diffractometer equipped with a two-dimensional area IP detector. Data collection was conducted using graphite-monochromated Mo–Kα radiation (λ = 0.71073 Å) and employed the oscillation scan technique with Δ*w* = 5° increment per image. The lattice parameters were accurately determined via least-squares methods based on all reflections where *F*^2^ > 2σ(*F*^2^). The integration of intensities, along with corrections for Lorentz and polarization effects and cell refinement, was carried out using the CrystalClear software by Rigaku/MSC, Inc., 2005. The structures were initially solved by direct methods with SHELXS-2013, which facilitated the localization of most of the heaviest atoms. The remaining nonhydrogen atoms were positioned from difference Fourier maps generated through successive cycles of full-matrix least-squares refinement on *F*^2^ using SHELXL-2013.^28^ All nonhydrogen atoms were refined using anisotropic displacement parameters, whereas the hydrogen atoms were modeled with common isotropic displacement factors and positioned using geometric restraints. The refinement process concluded with the final difference Fourier maps, which exhibited no significant residual peaks, indicating a clean and accurate structural model. Crystal data for **12**: C_8_H_10_O_3_Br_2_ H_2_O, crystal system, space group: monoclinic, *P*2_1_/*c*; (no: 14); unit cell dimensions: *a* = 11.470(4), *b* = 7.1440(3), *c* = 13.771(3) Å, α = 90, β = 97.976(59), γ = 90°; volume; 1117.5(2) Å^3^, *Z* = 4; calculated density: 1.973 g/cm^3^; absorption coefficient: 7.241 mm^–1^; *F*(000): 648; θ-range for data collection 1.8–25.2°; refinement method: full-matrix least-squares on *F*^2^; data/parameters: 1988/130; goodness-of-fit on *F*^2^: 1.086; Data completeness; 1.00, final *R*-indices [*I* > 2σ(*I*)]: *R*_1_ = 0.055, *wR*_2_ = 0.134; largest diff. peak and hole: 0.809 and −0.841 eÅ^–3^. Crystal data for **15**: C_15_H_15_O_5_Cl, crystal system, space group: monoclinic, *C*2/*c*; (no: 15); unit cell dimensions: *a* = 20.195(2), *b* = 10.1886(3), *c* = 13.990(2) Å, α = 90, β = 90.083(4), γ = 90°; volume; 2878.5(5) Å^3^, *Z* = 8; calculated density: 1.434 g/cm^3^; absorption coefficient: 0.284 mm^–1^; *F*(000): 1296; θ-range for data collection 2.9–25.5°; refinement method: full-matrix least-squares on *F*^2^; data/parameters: 2658/191; goodness-of-fit on *F*^2^: 1.286; final *R*-indices [*I* > 2σ(*I*)]: *R*_1_ = 0.089, *wR*_2_ = 0.161; largest diff. peak and hole: 0.286 and −0.380 eÅ^–3^.

## Data Availability

The data underlying this study are available in the published article and its Supporting Information.

## Supporting Information Available

^1^H NMR, ^13^C NMR, and mass spectrum of 6-(hydroxymethyl)-*N*-phenylcyclohex-3-ene-1-carboxamide; ^1^H NMR, ^13^C NMR, and mass spectrum of 5-bromo-6-hydroxyhexahydroisobenzofuran-1(3*H*)-one; and ^1^H NMR, ^13^C NMR, and mass spectrum of (1*R*,2*S*,4*S*,5*S*)-2-(((*tert*-butyldimethylsilyl)oxy)methyl)-4-hydroxy-6-oxabicyclo[3.2.1]octan-7-one (PDF)
