# Supplementary material for: Unusual Rearrangements in Cyclohex-3-ene-1-carboxamide Derivatives: Pathway to Bicyclic Lactones
Source: ACS Omega. 2024 May 14;9(21):22970–8. doi: 10.1021/acsomega.4c02183 (PMC11137725; doi:10.1021/acsomega.4c02183)
Supplement: Supplementary file 1 — ao4c02183_si_001.pdf [file ao4c02183_si_001.pdf]

# Supporting information for

## Unusual Rearrangements in Cyclohex-3-ene-1-carboxamide derivatives: Pathway to Bicyclic Lactones

Ozlem Gundogdu*[1,2], Sertan Aytaç[1,2], Ertan Şahin[2] and Yunus Kara*[2]

[1]*Ahi Evran University, Kaman Vocational School, Department of Food Technology, Kırsehir, Turkey*

[2]*Atatürk University, Faculty of Science, Department of Chemistry, Erzurum, Turkey*

## Table of contents

### Spectroscopic data

$^1$H NMR, $^{13}$C NMR and Mass spectrum of 6-(hydroxymethyl)-*N*-phenyl cyclohex-3-ene-1-carboxamide **(7b)**……2

$^1$H NMR, $^{13}$C NMR and Mass spectrum of (1*S*,6*R*)-6-(((tert-butyldimethylsilyl)oxy)methyl)-*N*-methylcyclohex-3-ene-1-carboxamide (**10a**)……3

$^1$H NMR, $^{13}$C NMR and Mass spectrum of (1*S*,6*R*)-6-(((tert-butyldimethylsilyl)oxy)methyl)-*N*-phenylcyclohex-3-ene-1-carboxamide (**10b**)……4

$^1$H NMR, $^{13}$C NMR and Mass spectrum of 5-bromo-6-hydroxyhexahydroisobenzofuran-1(3*H*)-one (**9**)……5

$^1$H NMR, $^{13}$C NMR and Mass spectrum of (3a*R*,5*S*,6*S*,7a*S*)-5,7a-dibromo-6-hydroxyhexahydroisobenzofuran-1(3*H*)-one (**12**)……6

$^1$H NMR, $^{13}$C NMR and Mass spectrum of (1a*R*,2a*S*,5a*R*,6a*S*)-hexahydrooxireno[2,3-*f*]isobenzofuran-3(1a*H*)-one (**14**) ……7

$^1$H NMR, $^{13}$C NMR and Mass spectrum of (3a*R*,5*R*,6*R*,7a*S*)-6-hydroxy-1-oxooctahydroisobenzofuran-5-yl 3-chlorobenzoate (**15**) ……8

$^1$H NMR, $^{13}$C NMR and Mass spectrum of (1R,2S,4S,5S)-2-(((*tert*-butyldimethylsilyl)oxy)methyl)-4-hydroxy-6-oxabicyclo[3.2.1]octan-7-one (**16**)……9

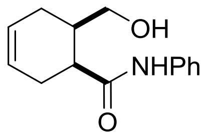


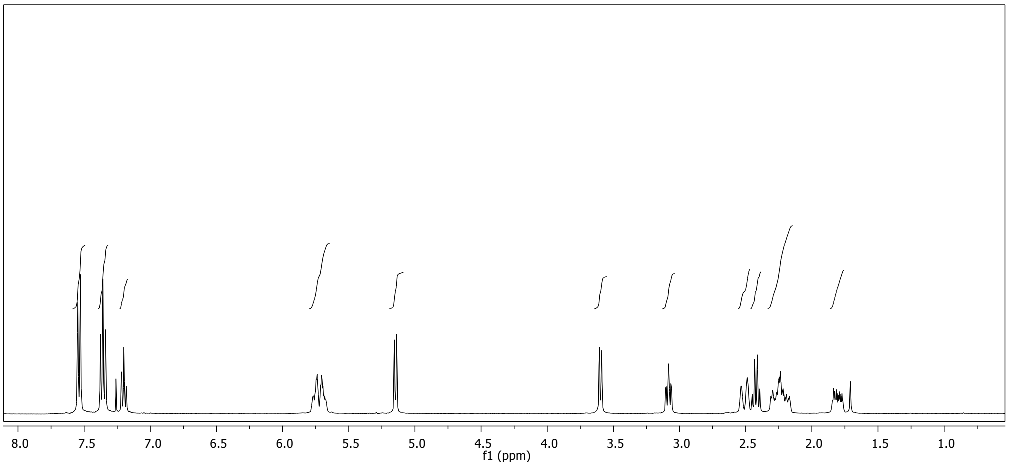


400 MHz $^{1}$H NMR spectrum of 6-(hydroxymethyl)-*N*-phenyl cyclohex-3-ene-1-carboxamide **(7b)** in $CDCl_3$

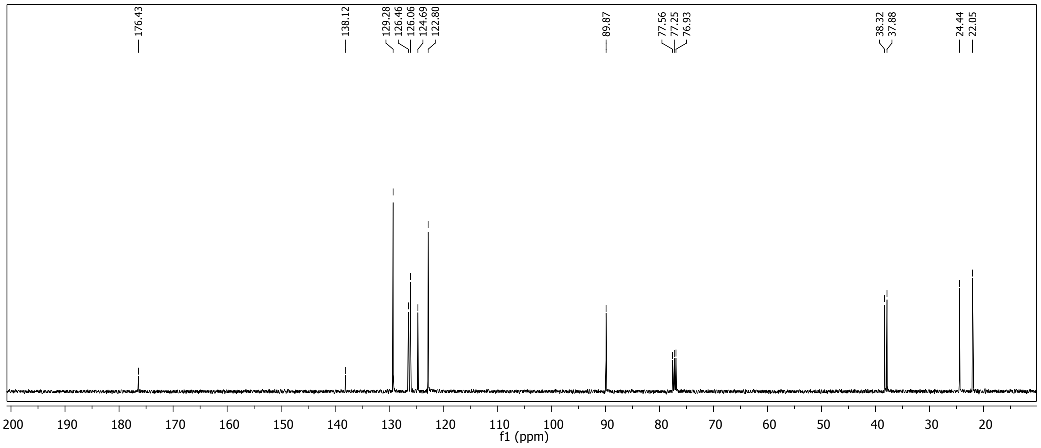


100 MHz $^{13}$C NMR spectrum of 6-(hydroxymethyl)-*N*-phenyl cyclohex-3-ene-1-carboxamide **(7b)** in $CDCl_3$

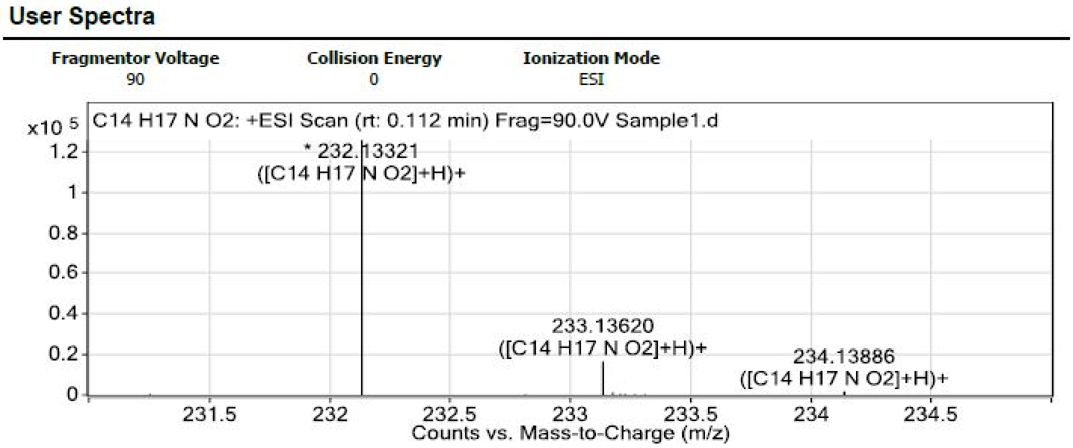


Mass spectrum of 6-(hydroxymethyl)-*N*-phenyl cyclohex-3-ene-1-carboxamide **(7b)**

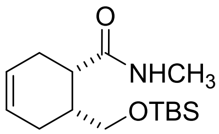


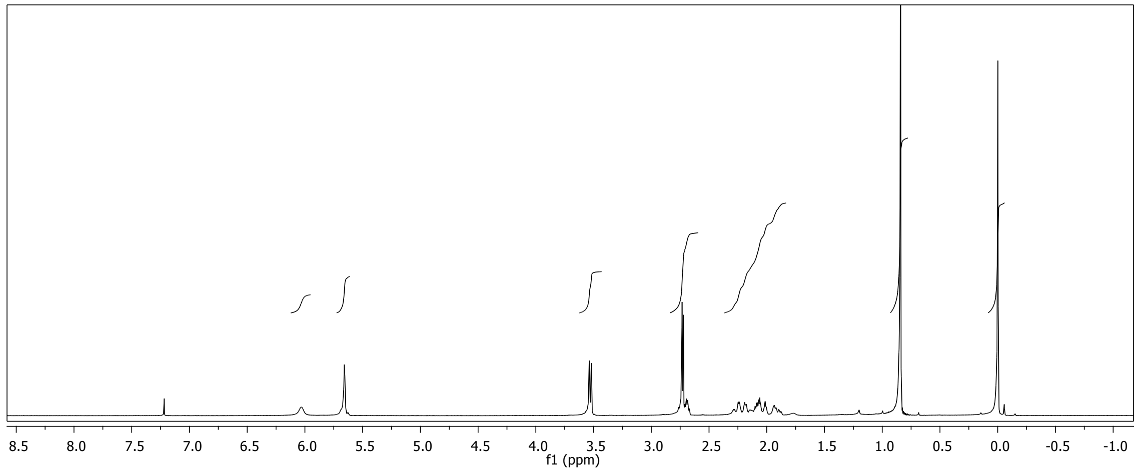


400 MHz $^{1}$H NMR spectrum of (1S,6R)-6-(((*tert*-butyldimethylsilyl)oxy)methyl)-*N*-methylcyclohex-3-ene-1-carboxamide **(10a)** in $CDCl_3$

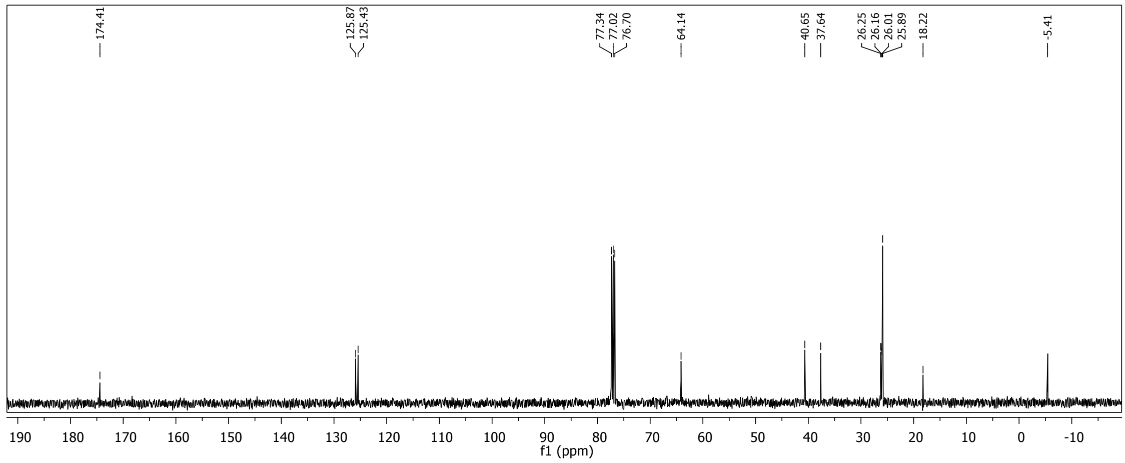


100 MHz $^{13}$C NMR spectrum of (1*S*,6*R*)-6-(((*tert*-butyldimethylsilyl)oxy)methyl)-*N*-methylcyclohex-3-ene-1-carboxamide **(10a)** in $CDCl_3$

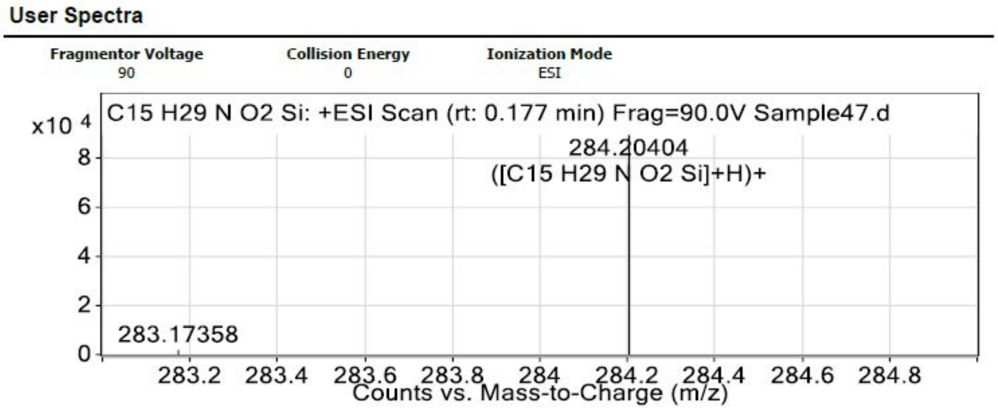


Mass spectrum of (1*S*,6*R*)-6-(((*tert*-butyldimethylsilyl)oxy)methyl)-*N*-methylcyclohex-3-ene-1-carboxamide **(10a)**

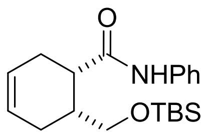


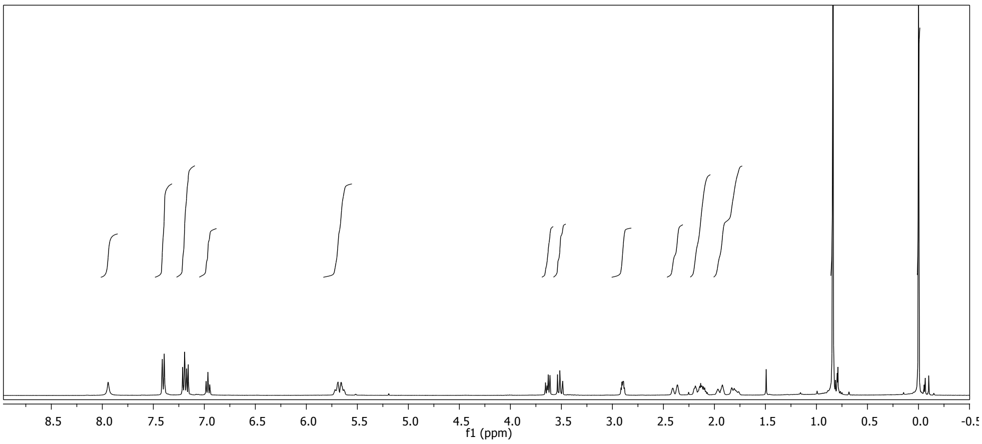


400 MHz $^{13}$C NMR spectrum of (1*S*,6*R*)-6-(((*tert*-butyldimethylsilyl)oxy)methyl)-*N*-phenylcyclohex-3-ene-1-carboxamide **(10b)** in $CDCl_3$

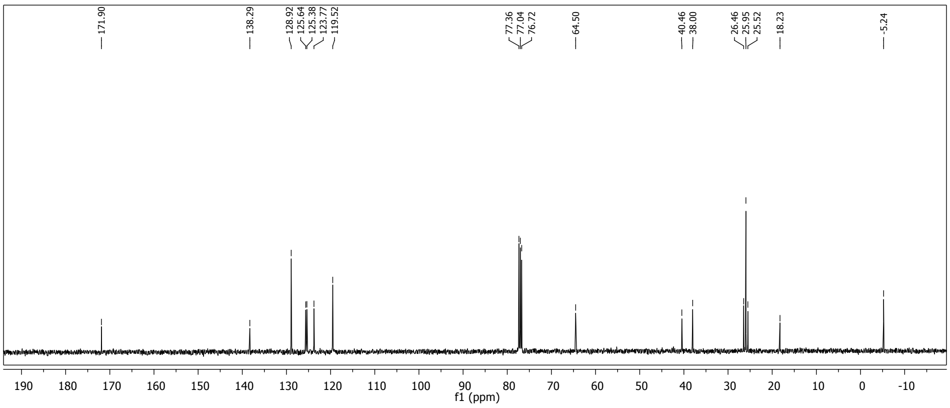


100 MHz $^{13}$C NMR spectrum of (1*S*,6*R*)-6-(((*tert*-butyldimethylsilyl)oxy)methyl)-*N*-phenylcyclohex-3-ene-1-carboxamide **(10b)** in $CDCl_3$

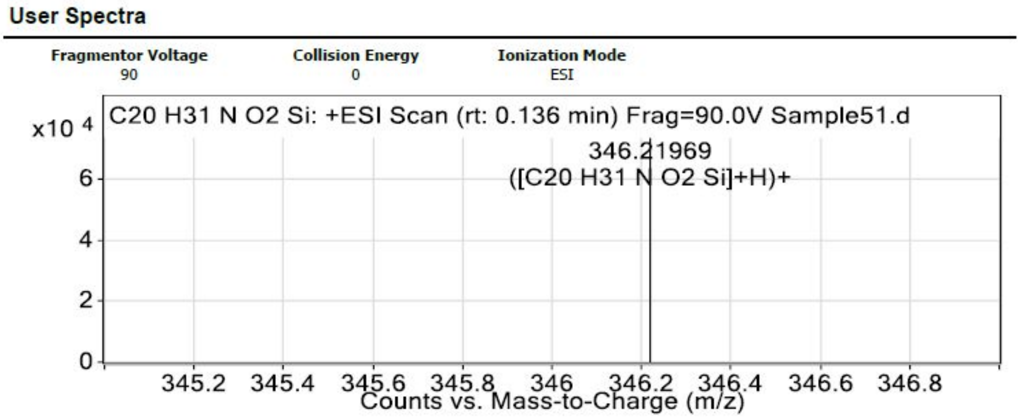


Mass spectrum of (1S,6R)-6-(((tert-butyldimethylsilyl)oxy)methyl)-N-phenylcyclohex-3-ene-1-carboxamide **(10b)**

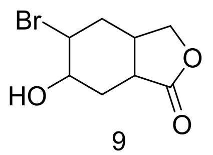


9

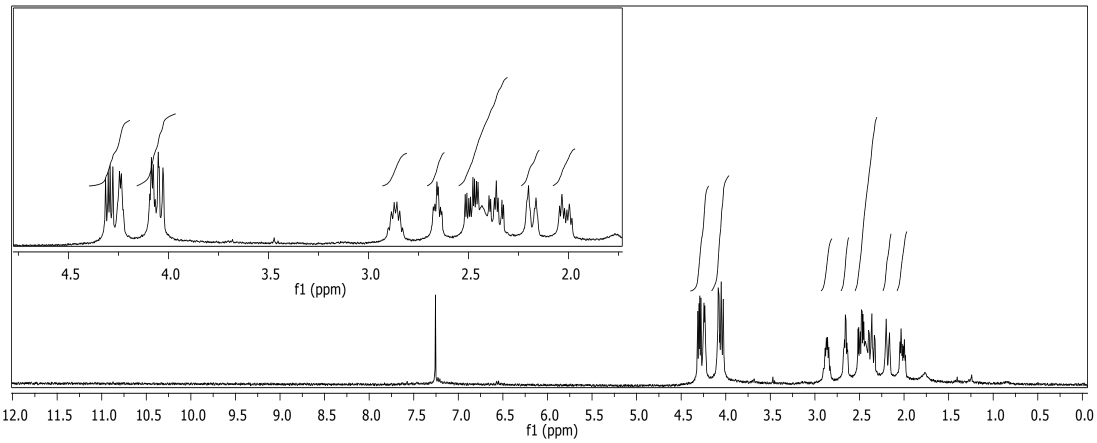


400 MHz $^{1}$H NMR spectrum of 5-bromo-6-hydroxyhexahydroisobenzofuran-1(3*H*)-one **(9)** in $CDCl_3$

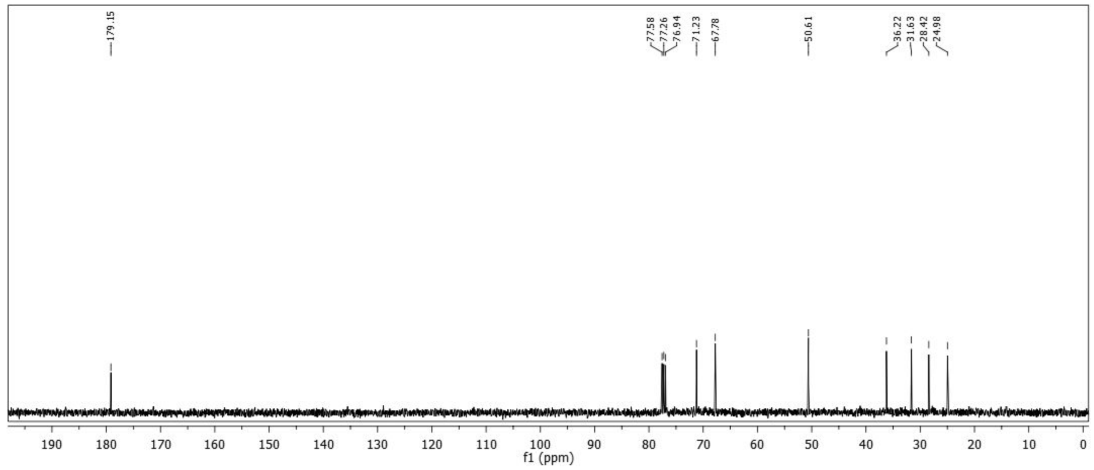


100 MHz $^{13}$C NMR spectrum of 5-bromo-6-hydroxyhexahydroisobenzofuran-1(3*H*)-one **(9)** in $CDCl_3$

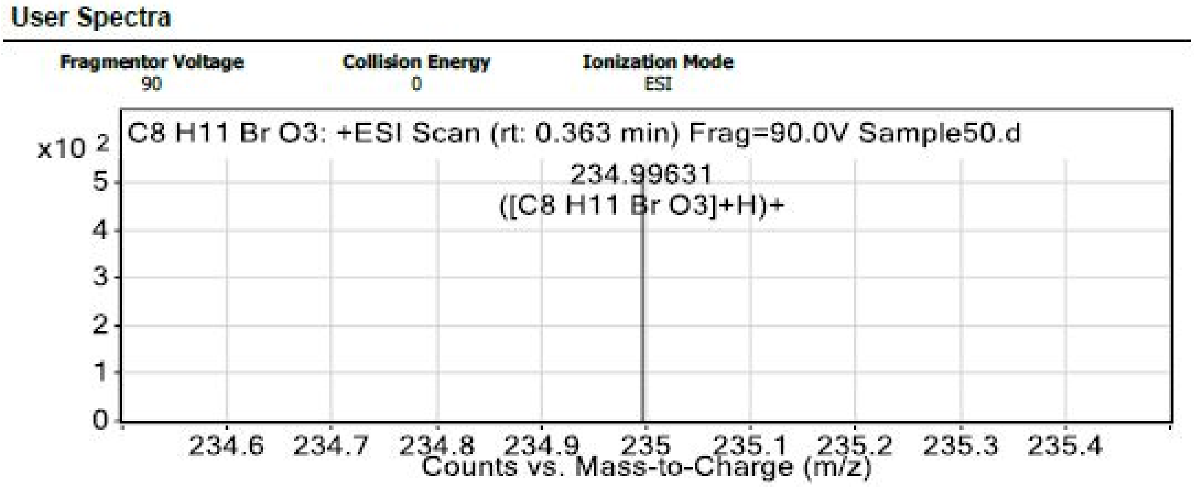


Mass spectrum of 5-bromo-6-hydroxyhexahydroisobenzofuran-1(3*H*)-one **(9)** in $CDCl_3$

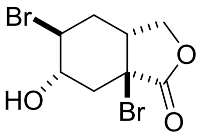


**12**

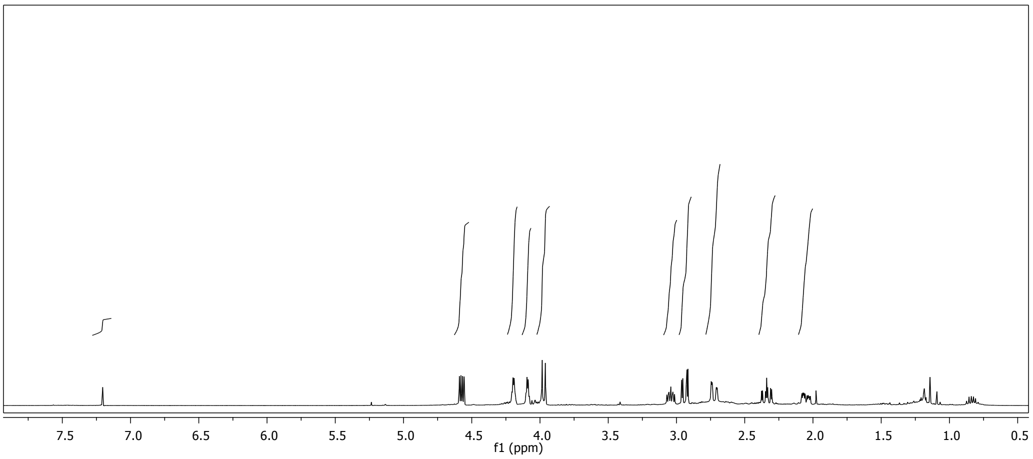


400 MHz $^1$H NMR spectrum of 5,7a-dibromo-6-hydroxyhexahydroisobenzo furan-1(3*H*)-one (**12**) in $CDCl_3$

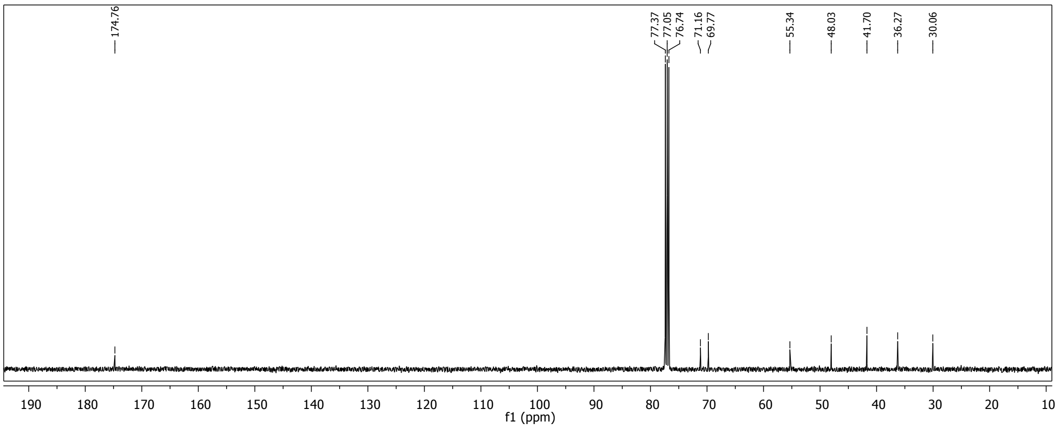


100 MHz $^{13}$C NMR spectrum of 5,7a-dibromo-6-hydroxyhexahydroisobenzo furan-1(3*H*)-one (**12**) in $CDCl_3$

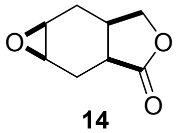


**14**

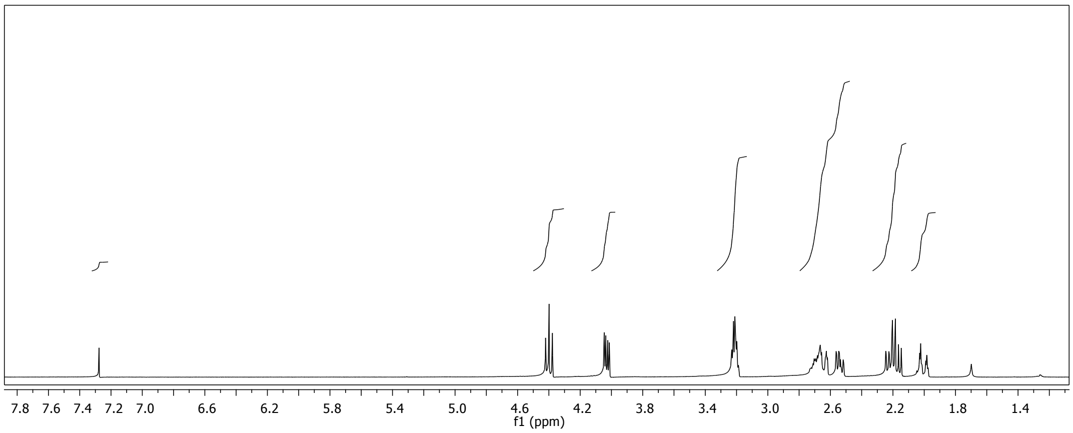


400 MHz $^{1}H$ NMR spectrum of hexahydrooxireno[2,3-*f*]isobenzofuran-3(1a*H*)-one **(14)** in $CDCl_3$

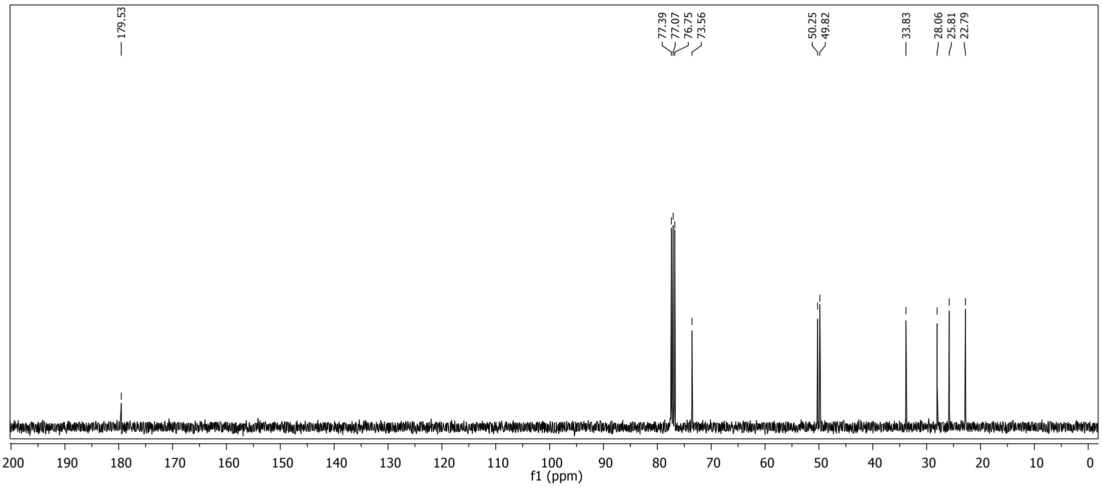


100 MHz $^{13}C$ NMR spectrum of hexahydrooxireno[2,3-*f*]isobenzofuran-3(1a*H*)-one **(14)** in $CDCl_3$

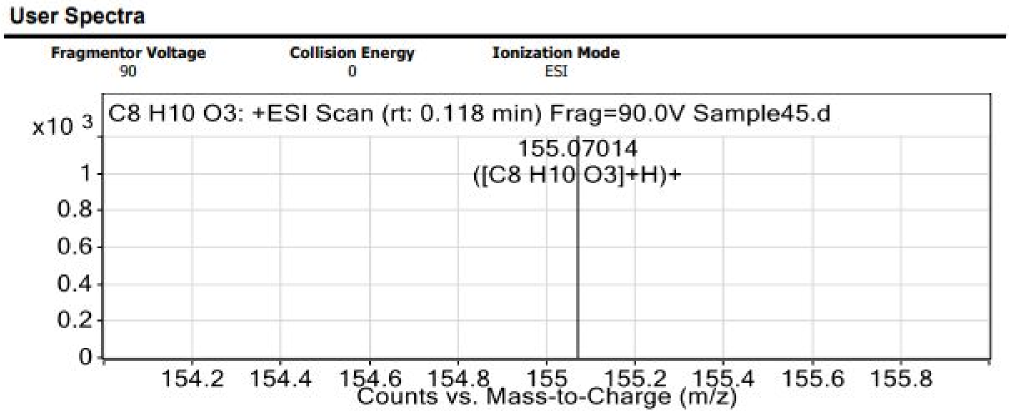


Mass spectrum of hexahydrooxireno[2,3-*f*]isobenzofuran-3(1a*H*)-one **(14)**

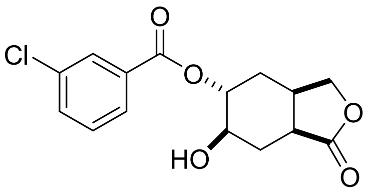


**15**

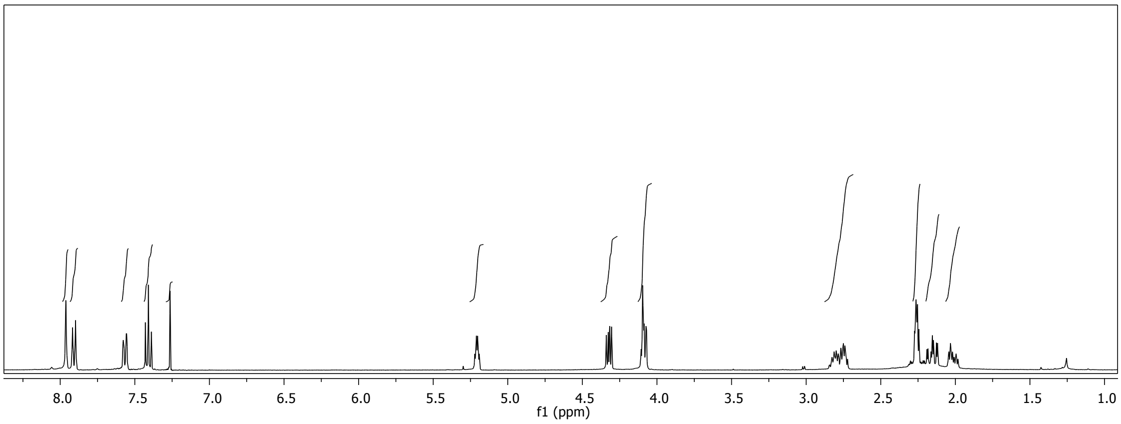


400 MHz $^{1}H$ NMR spectrum of 6-hydroxy-1-oxooctahydroisobenzofuran-5-yl 3-chlorobenzoate **(15)** in $CDCl_3$

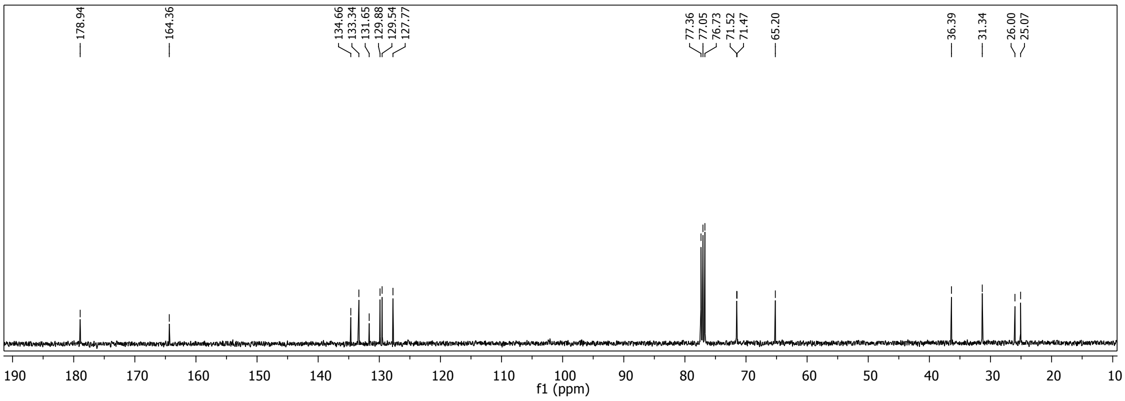


100 MHz $^{13}C$ NMR spectrum of 6-hydroxy-1-oxooctahydroisobenzofuran-5-yl 3-chlorobenzoate **(15)** in $CDCl_3$

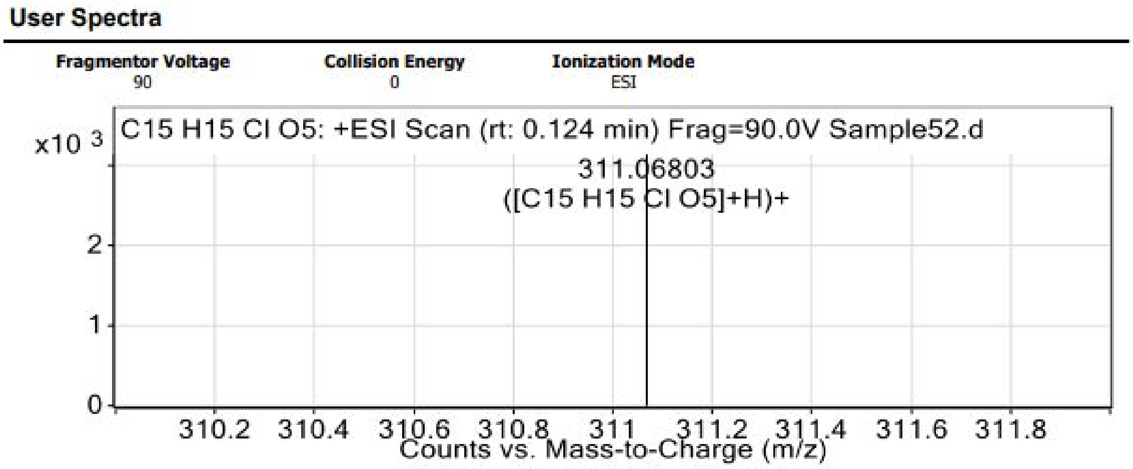


Mass spectrum of (3aR,5R,6R,7aS)-6-hydroxy-1-oxooctahydroisobenzofuran-5-yl 3-chlorobenzoate **(15)**

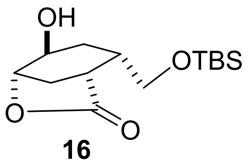


**16**

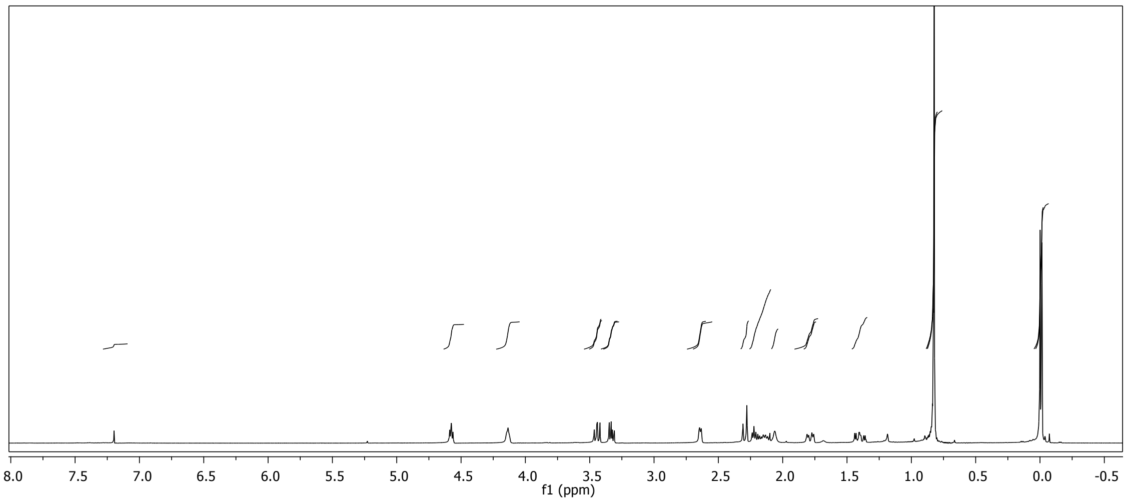


400 MHz $^{1}$H NMR spectrum of 2-(((*tert*-butyldimethylsilyl)oxy)methyl)-4-hydroxy-6-oxabicyclo[3.2.1]octan-7-one **(16)** in $CDCl_3$

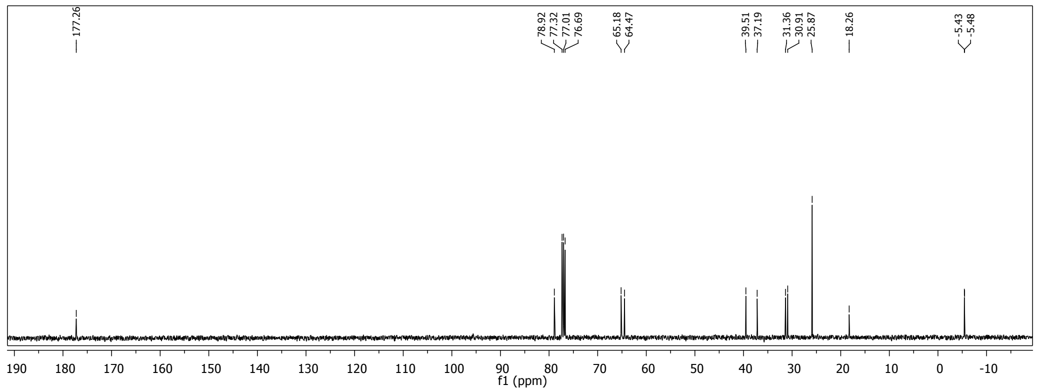


100 MHz $^{13}$C NMR spectrum of 2-(((*tert*-butyldimethylsilyl)oxy)methyl)-4-hydroxy-6-oxabicyclo[3.2.1]octan-7-one **(16)** in $CDCl_3$

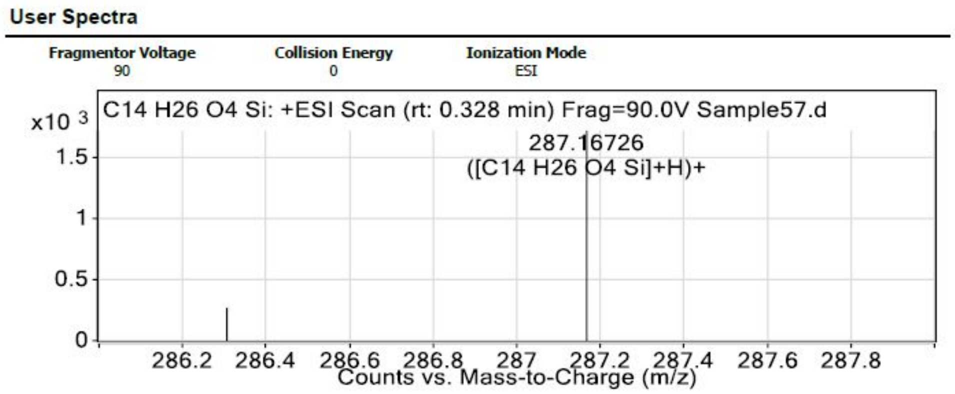


Mass spektrum of 2-(((tert-butyldimethylsilyl)oxy)methyl)-4-hydroxy-6-oxabicyclo[3.2.1]octan-7-one **(16)**
